# Dysregulated phosphate metabolism in autism spectrum disorder: associations and insights for future research

**DOI:** 10.1017/erm.2023.15

**Published:** 2023-06-13

**Authors:** Ronald B. Brown

**Affiliations:** University of Waterloo, School of Public Health Sciences, Waterloo, ON N2L 3G1, Canada

**Keywords:** Autism spectrum disorder, autism, cancer, dysregulated phosphate metabolism, epilepsy, gliosis, gluten-free casein-free diet, ketogenic diet, mitochondrial dysfunction, phosphate food additives, phosphate toxicity

## Abstract

Studies of autism spectrum disorder (ASD) related to exposure to toxic levels of dietary phosphate are lacking. Phosphate toxicity from dysregulated phosphate metabolism can negatively impact almost every major organ system of the body, including the central nervous system. The present paper used a grounded theory-literature review method to synthesise associations of dysregulated phosphate metabolism with the aetiology of ASD. Cell signalling in autism has been linked to an altered balance between phosphoinositide kinases, which phosphorylate proteins, and the counteracting effect of phosphatases in neuronal membranes. Glial cell overgrowth in the developing ASD brain can lead to disturbances in neuro-circuitry, neuroinflammation and immune responses which are potentially related to excessive inorganic phosphate. The rise in ASD prevalence has been suggested to originate in changes to the gut microbiome from increasing consumption of additives in processed food, including phosphate additives. Ketogenic diets and dietary patterns that eliminate casein also reduce phosphate intake, which may account for many of the suggested benefits of these diets in children with ASD. Dysregulated phosphate metabolism is causatively linked to comorbid conditions associated with ASD such as cancer, tuberous sclerosis, mitochondrial dysfunction, diabetes, epilepsy, obesity, chronic kidney disease, tauopathy, cardiovascular disease and bone mineral disorders. Associations and proposals presented in this paper offer novel insights and directions for future research linking the aetiology of ASD with dysregulated phosphate metabolism and phosphate toxicity from excessive dietary phosphorus intake.

## Introduction

The aetiology of autism spectrum disorder (ASD), a group of neurodevelopmental disorders that cause persistent social impairment with difficulties in communication and repetitive behavioural patterns, involves both genetic and environmental factors (Ref. 1). Prevalence of ASD rapidly increased from 1 out of 150 U.S. children in 2000, to 1 out of 68 children in 2012 and 1 out of 59 children in 2018 (Ref. 2). A recent systematic review and meta-analysis found that the male-to-female ratio of autism in children is approximately 3 to 1, which could be lower due to diagnostic gender bias that is less likely to diagnose females meeting ASD criteria compared to males (Ref. 3). By 2016, black children in the United States were 1.5 times more likely to be identified with ASD than white or Hispanic children, suggesting that ASD is associated with social determinants of health involving socio-economic status, housing, physical environment and racial discrimination (Ref. 4).

**Figure 1.**
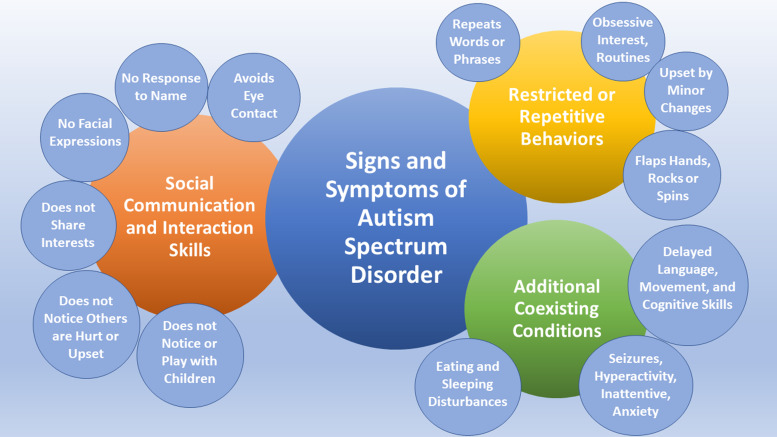
Signs and symptoms of autism spectrum disorder, based on American Academy of Pediatrics Council on Children With Disabilities (Ref. 5).

Figure 1 shows clinical signs and symptoms of ASD, based on information provided by the American Academy of Pediatrics Council on Children With Disabilities (Ref. 5). Age of onset of ASD symptoms ranges from the first year of infancy up to 2–3 years (Ref. 6). Adolescents with ASD have a higher risk of developing depression, anxiety and attention-deficit hyperactivity disorder (Ref. 7). With no known cause, treatments for ASD patients are mostly symptomatic to improve quality of life and daily functioning (Ref. 8).

Among potential environmental factors in ASD aetiology, dietary associations with ASD merit further investigations. In particular, studies of neurodevelopmental disorders from exposure to toxic levels of dietary phosphate are lacking. Dietary phosphorus in the form of inorganic phosphate (Pi) is an essential mineral that is regulated in the body by a sensitive network of hormones released by the kidneys, intestines, parathyroid glands and bone (Ref. 9). Dysregulation of phosphate metabolism can lead to the accumulation of excess phosphate in the body causing a condition known as phosphate toxicity. Exposure to phosphate toxicity can negatively impact almost every major organ system in the body, including the central nervous system, with possible implications for the aetiology of neurodevelopmental disorders in ASD.

## Method

The present narrative review used a grounded theory literature-review method (Ref. 10) to search, retrieve and analyse research findings using keywords related to dysregulated phosphate metabolism, phosphate toxicity and ASD. Grounded theory in this method replaces subjectivity and conjecture with a rigorous method to synthesise new information grounded in evidence. Through an iterative process of comparative analysis, findings from the research literature were developed into themes and formed into associated relationships, and additional keywords were searched to follow the trail of evidence and fill in knowledge gaps. The strength of this method's bottom-up inductive approach lies in discovering potential breakthrough knowledge grounded in findings from published literature (Ref. 11).

Nevertheless, ‘it is impossible to not be influenced by the background knowledge that one has’ (Ref. 10), and limitations of this method, as in most other research methods, include potential selection and information biases that influence data selection, analysis, interpretation and presentation of the research findings (Ref. 12). Findings may not be generalisable (external validity), and the researcher must successfully defend the work's internal validity under critical appraisal by peer experts. In the present paper, synthesised associations and proposals provide novel insights and directions for future research linking ASD aetiology and dysregulated phosphate metabolism.

## ASD, cancer and phosphate

A retrospective cohort study of over 8000 children and adolescents with autistic disorder estimated that cancer incidence within the cohort was 94% higher than the expected cancer incidence (standardised incidence ratio 1.94) (Ref. 13). A more recent population-based study of 2.3 million individuals from Nordic countries found that overall risk from any cancer increased in ASD, but only in individuals with comorbid intellectual disability and/or birth defects (Ref. 14), implying involvement of other causative factors such as chromosomal abnormalities related to mutations in PTEN (Phosphatase and Tensin Homolog), a tumour suppressor phosphatase (Ref. 14). Phosphatases and protein kinases are enzymes that modulate cellular proteins by catalysing the transfer of phosphate in opposing directions (Ref. 15). A protein kinase catalyses the transfer of phosphate from ATP or GTP to phosphorylate a protein while a phosphatase catalyses the transfer of phosphate from a phosphoprotein to a water molecule. Similar to cell signalling in cancer (Ref. 16), autism has been linked to an altered balance between phosphoinositide kinases and the counteracting effect of phosphatases in neuronal membranes (Ref. 17).

Phosphorus is a growth-rate limiting factor in tumorigenesis (Ref. 18). High dietary phosphate activates phosphoinositide 3-kinase (PI3 K) which phosphorylates Akt (protein kinase B) leading to activation of mTOR kinase that upregulates protein synthesis in cancer cell proliferation while suppressing apoptosis (Ref. 19). High dietary phosphate also suppresses the counteracting effect of PTEN on PI3 K (Ref. 20). A similar cell-proliferation effect is seen in autism and epilepsy as PI3 K activates tuberous sclerosis complex 1/2 (TSC1/2) which activates mTOR kinase (Refs 21, 22). PTEN mutations in autism and epilepsy also suppress counteraction of PI3 K, leading to tumours and neurodevelopmental disorders in PTEN hamartoma tumour syndrome (Ref. 23). A study of brain analyses found that white matter in PTEN-ASD patients had a specific overgrowth pattern, with poor white matter development, reduced processing speed, increased deficits in working memory and extensive intellectual limitations (Ref. 24). Results of these brain tissue analyses imply that high concentrations of Pi may accumulate in the white matter of PTEN-ASD patients, who make up approximately 20% of children with ASD (Ref. 25), and future studies should investigate Pi brain concentrations in PTEN-ASD patients.

Of relevance, phosphorus 31 magnetic resonance spectroscopy (MRS) was used in an earlier study to detect high concentrations of Pi in temporal lobe epilepsy (Ref. 26). More recently, increased Pi was detected in gliomas using MRS (Ref. 27), and dysregulated expression of the gene PHD-finger protein 3 (PHF3) in glioblastoma overlaps with similar dysregulated gene expression of PHF3 in autism (Ref. 28). Increased benign glioma risk of the central nervous system in children is also associated with neurofibromatosis type 1 (NF1) (Ref. 29), and autistic behaviour is elevated in children with NF1 (Ref. 30).

Feasibly, as in cancer, future research may show that high dietary phosphate associated with phosphate toxicity activates PI3 K and causes PTEN mutations in autism and epilepsy, leading to overgrowth and defective neuronal connectivity. Of relevance, phosphoric acid used as a common food additive was found to cause genotoxic damage to DNA in human lymphocytes (Ref. 31), which is an epigenetic factor that could increase PTEN mutations. Furthermore, lower urinary excretion of phosphoric acid was found in children with autism compared to controls (Ref. 32), possibly indicating higher retention of phosphoric acid in body tissue with greater genotoxic damage in children with autism. More research is needed in the role of dysregulated phosphate metabolism in PTEN mutations in ASD.

## ASD, dietary phosphate and gliosis

Infants who have been breastfed for long periods have better cognitive development and lower risk of autistic traits (Ref. 33). Phosphorus is about six-times lower in human breast milk (15 mg per 100 ml) than in cow milk (Ref. 34), and phosphorus plasma levels were lower and calcium levels higher in breastfed newborn babies compared to newborn babies fed unmodified cow milk (Ref. 35). In infancy, high phosphorus, sodium, potassium, protein and chloride intake from inappropriate consumption of whole cow milk places a burden on developing kidneys' solute load (Ref. 36). These findings suggest that breastfeeding is associated with lower risk of renal burden and reduced incidence of dysregulated phosphate metabolism potentially related to autistic traits.

Casein in cow milk, a phosphoprotein with phosphate groups bonded to amino acid side groups, makes up about 80% of cow milk protein (Ref. 37). Accordingly, dietary patterns that reduce casein also reduce phosphorus, which may account for many of the suggested benefits of casein-free diets in children with ASD (Ref. 38). Although gluten protein in grain does not contain phosphorus, bread and baked goods are often high in phosphorus and phosphate additives (Ref. 39). While a negative effect from gluten ingestion remains unproven in ASD, gluten-free diets are suggested to have beneficial effects in ASD (Ref. 40), which might be attributed to reductions in overall intake of phosphorus in bread and baked goods.

The rise in ASD has been suggested to originate in changes to the gut microbiome from increasing consumption of additives in processed food. Abdelli *et al*. found that exposing human neuron stem cells to the common food preservative propionic acid increased inflammatory responses and gliosis (excessive proliferation of fibrous support cells in the central nervous system) as occurs in neuro-circuitry dysfunction in ASD (Ref. 41). Interestingly, the researchers also found that propionic acid decreased levels of PTEN by about 50% compared to controls, which allowed levels of activated p-Akt (phosphorylated Akt) to increase and stimulate glial cell proliferation. Abdelli *et al*. also noted decreased neurite outgrowth in neuronal cells, which differentiate into dendrites or axons, and decreased axonal expansion was also noted which the researchers attributed to a physical barrier caused by excessive glia. The researchers proposed that dysregulated outgrowth and expansion explained disrupted communication between neuronal cells reported in ASD.

**Figure 2.**
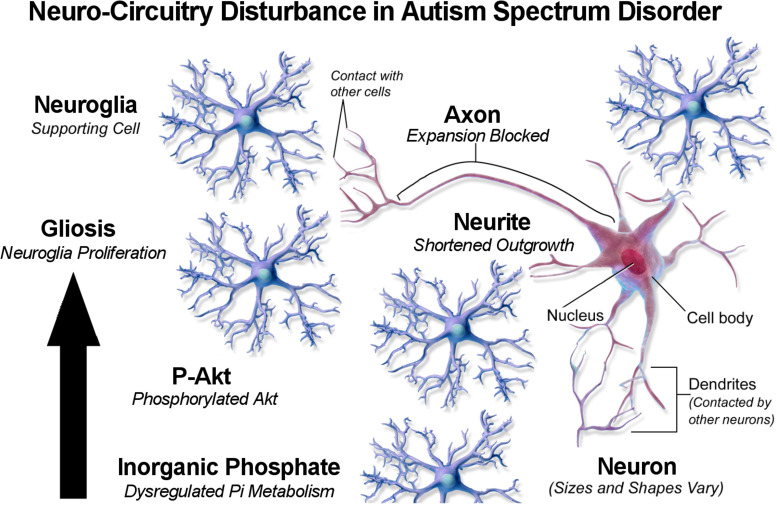
Neuro-circuitry disturbance in autism spectrum disorder. Based on https://upload.wikimedia.org/wikipedia/commons/7/73/Blausen_0672_NeuralTissue.png.

Figure 2 is a schematic diagram proposing that Pi from dysregulated phosphate metabolism phosphorylates Akt, leading to gliosis and neuro-circuitry disturbance in ASD. Future research should test this mechanism with in vitro experiments that compare glial cell proliferation in cultures exposed to concentrations of Pi, as well as in vivo experiments comparing levels of dietary phosphate and gliosis in lab animals. Although rodents are commonly used to model genetic mutations in autism, limited use of non-human primates comes closer to matching autistic behaviour in humans (Ref. 42).

## ASD, dysregulated phosphate and immune/neuroinflammatory responses

Abdelli *et al*. measured immune responses and neuroinflammation in human neural stem cells from exposure to propionic acid, including increased levels of inflammatory cytokines such as tumour necrosis factor alpha, with smaller increases in the anti-inflammatory cytokine interleukin 10 (Ref. 41). Rodriguez and Kern proposed that reducing neuroinflammation and microglial activation could lead to improvements in neurodevelopment outcomes in people with ASD (Ref. 43).

Feasibly, immune responses related to neuro-circuitry disturbances in ASD may be caused by dysregulated phosphate. The following text compares abnormalities of natural killer cells (NK-cells), T-cells, B-cells, macrophages and monocytes in patients with ASD (Ref. 44) with similar immune responses in conditions involving dysregulated phosphate. Although NK-cell proliferation was reported to increase in ASD, the NK-cells had reduced functioning ability (Ref. 45). Normally, NK-cells regulate neuron and glia functions, including proliferation and neurite outgrowth (Ref. 46), implying that NK-cell dysfunction and neuro-circuitry disturbances in ASD may share a common cause from exposure to phosphate toxicity. Of relevance, NK-cells are dysfunctional in end-stage kidney disease patients receiving dialysis therapy (Ref. 47), which is a procedure that helps control dysregulated phosphate (Ref. 48).

T-cell abnormalities in ASD include reduced proliferation of T-helper cells (Th) and reduced regulatory T cells (Treg) with upregulation of Th2 cells (Ref. 44). Interestingly, Th2 cells are involved in hypersensitivity to food antigens (Ref. 49) which may include hypersensitivity from exposure to excessive dietary phosphate. Of relevance to increased skin sensitivity in ASD (Ref. 50), high serum phosphate is associated with skin itching—pruritus (Ref. 51), and pruritus is associated with increased Th2 cytokines (Ref. 52). T cell exhaustion also occurs in kidney failure (Ref. 53), which is associated with harmful levels of serum phosphate (Ref. 54).

Children with ASD also have higher levels of pro-inflammatory B-cells [34]. B-cell infiltrates that occur in renal interstitial inflammation (Ref. 55) are potentially related to B-cell infiltrates in kidney burden from phosphate toxicity (Ref. 56). Macrophage and monocyte immune responses are also common in ASD (Ref. 44) and in conditions with dysregulated phosphate such as kidney disease. For example, accumulation of macrophages and monocytes in renal tissue causes inflammation and is associated with renal tissue damage, scarring and reduced kidney function (Ref. 57).

Dysregulated Pi in chronic kidney disease (CKD) also shares many other enzymes, immune responses and inflammatory markers with ASD. Compared to children with normal development, children with ASD were found to have elevated protein expression for IL-1*β*, IL-4, IL-9, interferon gamma (IFN-*γ*), Janus kinase 1 (JAK1), phosphorylated JAK1 (pJAK1), signal transducer and activator of transcription 5 (STAT5) and pSTAT5 (Ref. 58). Similarly, patients with diabetic nephropathy, a leading cause of end-stage renal disease with dysregulated Pi, have increased expression of IL-1*β* and IL-4 (Ref. 59), and overproduction of IL-9 and IFN-*γ* was found in patients with acute glomerular injury of the kidneys (Ref. 60). Additionally, JAK1 expression is enhanced in diabetic nephropathy (Ref. 61), phosphorylated JAK1 stimulates production of large amounts of FGF23, a regulator of phosphate metabolism (Ref. 62), abnormal amounts of STAT5 are expressed in autosomal dominant polycystic kidney disease (Ref. 63), and pSTAT5 is increased in focal segmental glomerulosclerosis of the kidneys (Ref. 64).

Compared to children with normal development, children with ASD showed elevated expression of chemokine receptors in CD4 + T cells, including chemokine receptor 2 + (CXCR2 + ), CXCR3 + , CXCR5 +  and CXCR7 + and C-C motif chemokine receptor 3 + (CCR3 + ), CCR5 + , CCR7 +  and CCR9+ (Ref. 65). Correspondingly, chemokine receptors CXCR1-7 are highly expressed in clear cell renal cell carcinoma (Ref. 66), CCR3 is upregulated in renal cell carcinoma (Ref. 67), CCR5 + contributes to the pathogenesis of CKD (Ref. 68), and children with CKD have increased expression of CCR7 and other chemokine receptors (Ref. 69). Furthermore, CCR9 + is associated with food allergens (Ref. 70), which may include reactions to exposure to excessive dietary phosphate.

Nuclear factor-erythroid 2 related factor 2 (Nrf2), a transcription factor that protects immune cells against inflammation and oxidation, was reduced in children with autism (Ref. 71). Similarly, impaired production of Nrf2 in CKD increases oxidation and inflammation leading to nephritis (Ref. 72). Severity of symptoms in children with autism is associated with upregulation of IL-6 receptors and IL-17A in CD4 + T cells (Ref. 73), and upregulated IL-17A expression in neutrophils increases inflammation and oxidation in children with autism (Ref. 74). Likewise, IL-17A plays a central role in kidney disease pathogenesis (Ref. 75) while IL-6 increases expression of FGF23 in acute and CKD (Ref. 76). Autism in children is also associated with elevated expression of IL-16 in CD4 + , CD8 + , CD14 + , CCR3 +  and CXCR7 + cells (Ref. 77). Similarly, IL-16 contributes to inflammation of the kidneys which can lead to impaired kidney function (Ref. 78). Furthermore, immune dysfunction in children with autism is associated with upregulated T cell immunoglobulin and mucin domain 3 (TIM-3) (Ref. 79), and overexpression of TIM-3 is associated with severe kidney inflammation (Ref. 80).

Research is needed to explore common mechanisms that explain how dysregulated phosphate metabolism may contribute to immune and inflammatory responses in both kidney disease and ASD. Other environmental factors and toxic conditions can also contribute to ASD—nevertheless, evidence implicating dysregulated phosphate metabolism in ASD warrants further investigation.

## ASD, phosphate additives and ultraprocessed food

U.S. consumption of ultra-processed foods, defined as industrially made, ready-to-eat or heat-and-serve products that contain food additives and generally lack whole foods, has continuously increased over the past two decades (Ref. 81), concurrent with increases in ASD prevalence. Children with ASD often prefer highly processed foods with phosphate additives, including chicken nuggets, pizza, macaroni with cheese, breakfast cereals, pancakes, waffles, hot dogs, crackers and bread, with less preference for fresh produce (Ref. 82). Compared to children with typical development, children with ASD were found to consume approximately 20–30% more ultra-processed food (Ref. 83). Of relevance, consumption of ultraprocessed food high in inorganic phosphates is associated with renal function decline in older adults (Ref. 84).

Additionally, a recent systematic review and meta-analysis found a 58% increased risk of obesity associated with ASD in children compared to controls (Ref. 85). Higher prevalence of childhood obesity in ASD may be related to higher energy intake and weight gain caused by ultra-processed food intake, as demonstrated in a controlled study of ultra-processed food consumed by adults with obesity (Ref. 86). Maternal intake of ultra-processed food is also associated with increased obesity in offspring (Ref. 87). Furthermore, a national cross-sectional study found that ultra-processed food consumption is greater among younger aged, lower income and less educated Americans (Ref. 88), and more research is needed to compare these demographic findings with ASD prevalence. Because neurodevelopment in newborns is critically influenced by maternal prenatal diet, which is suggested to play a role in ASD aetiology (Ref. 89), research should investigate whether younger mothers with less education, lower income, obesity and greater consumption of ultra-processed food have a higher risk of ASD in their offspring.

Many sugar-sweetened beverages (SSBs), such as colas, contain phosphoric acid, and increased consumption of SSBs is associated with stronger and more difficult emotional problems in ASD (Ref. 90). In addition, baking powder, chocolate, cocoa and beer are common sources of dietary phosphate that may contribute to excessive phosphate intake (Ref. 91). A 2019 national survey of food items in Finland (representative of Europe) found phosphate additives in 36% of sampled foods, and 17 different phosphate additives were observed overall (Ref. 92). Phosphorus-containing food additives are listed in Table 1.

**Table 1.** Phosphorus-containing food additives

Inorganic phosphates	Organic phosphates
*Orthophosphate*	*Starch phosphates*
Phosphoric acid	Monostarch phosphate
Sodium phosphate, mono-, di-, tri-	Distarch phosphate
Potassium phosphate, mono-, di-, tri-	Phosphated distarch phosphate
Calcium phosphate, mono-, di-, tri-	Acetylated distarch phosphate
*Magnesium phosphates*	Hydroxypropyl distarch phosphate
Magnesium phosphate, mono-, di-	**Natural phosphorus-containing additives**
*Pyrophosphates*	*Ribonucleotides*
Sodium diphosphate, di-, tri-, tetra-	Guanylic acid
Tetrapotassium diphosphate	Disodium guanylate
Dicalcium diphosphate	Dipotassium guanylate
Calcium dihydrogen phosphate	Calcium guanylate
Magnesium dihydrogen phosphate	Inosinic acid
*Triphosphates*	Disodium inosinate
Pentasodium phosphate	Dipotassium inosinate
Pentapotassium diphosphate	Calcium inosinate
*Polyphosphates*	Calcium-5′-ribonucleotides
Sodium polyphosphate	Disodium-5′-ribonucleotides
Potassium polyphosphate	*Other*
Sodium calcium polyphosphate	Lecithin
Calcium polyphosphate	Ammonium phosphatide
*Other*	Riboflavin-5′-phosphate
Sodium aluminium phosphate	

Based on Tuominen *et al*. (Ref. 92).

Sodium aluminium phosphate is commonly added to baking powder and processed cheese (Ref. 93). Excessive environmental exposure to aluminium within the body causes aluminium ions to bind with phosphate, which interferes with phosphorylation and dephosphorylation mechanisms in kinase and phosphatase enzymes, and contributes to mitochondrial dysfunction, microglia activation and immune and neuroinflammatory responses (Ref. 94). Compared to controls, greater levels of aluminium in brain tissue have been detected in autism and neurodegenerative diseases (Ref. 95), and intracellular aluminium in autism is concentrated within microglial cells and other non-neuron cells of brain tissue (Ref. 96).

In addition to higher dietary phosphate intake, average concentrations of urinary phosphorus excretion are 34% lower in children with ASD (Ref. 97), suggesting positive net phosphate absorption and tissue storage in ASD. Importantly, serum phosphate levels do not always accurately reflect tissue Pi storage levels (Ref. 98), and abnormal amounts of phosphate shifted from serum to tissue storage could explain hypophosphatemia found in some children with ASD compared to healthy children (Ref. 99). More studies are needed using phosphorus 31 MRS for detection of phosphate tissue storage in autism during brain development (Ref. 100).

Additionally, dietary fat is free of phosphorus, and lower phosphorus intake levels may explain benefits in ASD from high-fat ketogenic diets (Ref. 101). Ketogenic diets are effective in reducing or preventing seizures in epilepsy (Ref. 102), and ASD is associated with epilepsy (Ref. 103), implying that both ASD and epilepsy may share a common pathophysiological cause related to dysregulated phosphate metabolism and phosphate toxicity.

Evidence suggests that excessive dietary protein intake above optimal levels for neurodevelopment can be as harmful as deficient protein intake in perinatal care (Ref. 104). Of relevance, each gram of protein from dietary sources is directly related to approximately 12–14 mg phosphorus (Ref. 105). Consequently, dietary protein intake levels above optimal amounts for neurodevelopment are likely to involve excessive amounts of phosphorus with increased risk of dysregulated phosphate metabolism.

Higher ASD prevalence in males is consistent with higher mean dietary phosphorus intake in U.S. male children aged 2–11 years compared to females, with significantly higher phosphorus intake in male adolescents aged 12–19 years (Ref. 106). Interestingly, some children with ASD show improvements in behaviour during fever (Ref. 107). Coincidently, tumours also regress following fever (Ref. 108), and fever is associated with reduced appetite (Ref. 109), inferring lower food intake. Accordingly, reduction in dietary phosphate intake associated with fevers may act as a mediating factor that temporarily improves behaviours in ASD and may reduce tumours as lower phosphate levels limit the rate of cancer cell growth.

## ASD, diabetes and dysregulated phosphate

Large studies have found that ASD is associated with increased prevalence of type 1 and type 2 diabetes mellitus (Ref. 110). Of relevance, neuronal degeneration and other complications in diabetes are associated with dysregulated phosphate metabolism (Ref. 111). These findings infer that dysregulated phosphate metabolism is a pathophysiological mechanism common to diabetes mellitus and neuronal degeneration in ASD. Maternal diabetes in rats induces autism-like behaviour in offspring (Ref. 112). In humans, maternal obesity comorbid with diabetes is associated with a greater risk of ASD in offspring compared to obesity or diabetes alone (Ref. 113). This finding implies that high dietary phosphate intake in obesity may interact with dysregulated phosphate metabolism in diabetes, leading to greater associated risk of ASD. Further research is needed to confirm an interactive relationship in ASD between obesity and diabetes mediated by excessive phosphate intake.

Prevalence of neurodevelopmental disorders, including ASD, is higher in children with type 1 diabetes, and diabetic nephropathy and retinopathy are also higher in type 1 diabetic children with neurodevelopment disorders (Ref. 114). Of relevance, children with ASD have a higher risk of ophthalmologic diagnosis (Ref. 115) and 25% of adults with ASD were found to have CKD (Ref. 116), providing further support for dysregulated phosphate metabolism as a common pathophysiological cause in diabetic complications and ASD. Furthermore, low muscle tone or hypotonia is an established marker of individuals with ASD (Ref. 117). Similarly, hypotonia affects children with dysregulated phosphate metabolism in CKD (Ref. 118), and hypotonia is associated with increased mortality in patients with renal failure receiving dialysis therapy for hyperphosphatemia (Ref. 119).

## ASD, mitochondrial dysfunction and tauopathy

Mitochondrial dysfunction is associated with pathogenesis of ASD, and occurs in up to 80% of children with ASD (Ref. 120). Coincidentally, mitochondrial dysfunction is associated with dysregulated phosphate metabolism in the pathogenesis of diabetes (Ref. 111) and Parkinson's disease (Ref. 121). Precipitates of calcium phosphate collect within the mitochondria matrix of pancreatic beta cells and substantia nigra cells that produce insulin and dopamine in diabetes and Parkinson's disease, respectively, which interfere with the function of complex I in the electron transfer chain during oxidative phosphorylation. The result of mitochondrial dysfunction in cells includes reduced ATP biosynthesis, apoptosis and cell death. Importantly, the majority of children with autism in an exploratory controlled study had below-normal values of complex 1 activity (Ref. 122). Research is needed to examine the pathophysiologic effect of dysregulated phosphate metabolism, phosphate toxicity and calcium phosphate precipitation in mitochondrial dysfunction affecting neuronal cells of individuals with ASD.

In tauopathies associated with neurodegenerative diseases like Alzheimer disease and Parkinson's disease (Ref. 123), hyperphosphorylation of tau, a protein that assists in neuron cell maturation and cytoarchitecture, causes microtubule dysfunction which is linked with tau aggregation and neurofibrillary tangles (Ref. 124). Autism-like behaviours have been linked to increased levels of frontotemporal lobe tau and neurofibrillary pathology in late-life dementia (Ref. 125). Genetically lowering or removing tau in mouse models prevented or reduced key features of ASD, including abnormally enlarged head size, epilepsy and overactivation of the PI3 K/Akt/mTOR signalling pathway, which resulted in disinhibition of PTEN (Ref. 126). Drugs that interfere with tau phosphorylation are proposed to treat tauopathies (Ref. 124), but increased attention should focus on dietary interventions that reduce excessive phosphate intake, which may act as a rate-limiting factor to reduce tau hyperphosphorylation in the aetiology of neurodegenerative diseases and neurodevelopmental diseases like ASD. Importantly, hyperphosphorylation of tau is associated with glial-tau pathology in glial cells (Ref. 127), further implicating hyperphosphorylation as a cause of excessive glial cell proliferation and gliosis, which disrupts neuro-circuitry in ASD.

## ASD, vitamin D, bone disorders and cardiovascular disease

The bioactive form of vitamin D, 1,25(OH)_2_D_3_, also known as calcitriol, is synthesised by the kidneys from the storage form of vitamin D, 25-hydroxy vitamin D, 25(OH)D_3_ (Ref. 9). Calcitriol increases intestinal absorption of Pi during digestion, raising serum phosphorus levels as needed. To reduce excessive levels of serum Pi, the kidneys produce less bioactive vitamin D, which decreases phosphorus absorption in the intestines. Low levels of 25(OH)D_3_, are common in ASD (Ref. 128), and lower serum levels of calcitriol and calcium were also found in ASD compared to normal controls (Ref. 129)—a potential biomarker indicating a kidney response in ASD that attempts to reduce serum Pi by lowering phosphate intestinal absorption when dietary phosphate intake is excessive.

Other endocrine hormones downregulate serum Pi by increasing renal phosphaturia, including fibroblast growth factor 23 (FGF23) released from bone and parathyroid hormone (PTH) released from the parathyroid glands (Ref. 9). A recent case study reported elevated serum PTH and serum phosphate in a 14-year old boy with ASD having hypocalemic seizures following a prolonged diet of processed snack foods (Ref. 130). Researchers also found structural changes in the frontal lobes associated with increased FGF23, likely in response to high levels of Pi which could also explain increased vascular calcification and microangiopathic lesions found in the brain leading to white matter loss (Ref. 131). In general the brain's frontal lobes control social and cognitive behaviour, and frontal cortex damage is known to lead to the onset of persistent and repetitive behaviour, insistence for sameness, and impulsive behaviours which are clinical features of ASD (Ref. 132). Interestingly, low serum levels of basic fibroblast growth factor, fibroblast growth factor 2 (FGF2), which regulates neurodevelopment, were found in children with ASD (Ref. 133). Future research is needed to investigate how dysregulated Pi impacts high FGF23 and low FGF2 levels in young children with ASD.

Odds of hip fractures in a case-control study were higher in children and adults with ASD compared to non-ASD individuals, and odds of fractures of the forearm and spine were greater in women with ASD (Ref. 134). Compared to typically developing children, a meta-analysis found that children with ASD had up to 13% lower bone mineral density of the total body, which is associated with increased risk of fractures (Ref. 135). Osteoporosis and fracture are also associated with dysregulated phosphate metabolism in CKD-mineral and bone disease (CKD-MBD) (Ref. 136), suggesting a common cause with fractures in ASD.

Bone mineral disorders related to dysregulated phosphate metabolism also impact poor oral health and periodontal disease, and dental plaque associated with gingivitis and periodontitis often forms from excessive calcium phosphate in saliva (Ref. 137). A meta-analysis in children and young adults with ASD found a pooled prevalence of approximately 70% for periodontal disease (Ref. 138), implicating phosphate toxicity in ASD as a potential pathophysiological factor in poor oral health.

Autism is also associated with endothelial dysfunction of the arterial system, which contributes to disruption of neurovascular coupling mechanisms in neurodevelopment (Ref. 139). In another potential link between ASD and dysregulated phosphate metabolism, hyperphosphatemia has a deleterious effect on endothelial dysfunction (Ref. 140). Of relevance, risk factors for cardiovascular disease (CVD) are increased in CKD, and the primary treatment goal in CKD is control of phosphate load (Ref. 141). Increased risk for heart failure and atrial fibrillation in CKD (Ref. 142) overlaps with similar risk for heart failure and atrial fibrillation in ASD (Ref. 143). Moreover, a higher risk of death in people with coronary disease was associated with higher serum phosphate levels (Ref. 144). Even people with normal renal function were found to have an increased risk of cardiovascular events associated with higher serum phosphate (Ref. 145). Although many environmental factors that contribute to ASD pathogenesis are shared with kidney disease, studies are lacking that investigate the association of paediatric kidney disease with ASD in children (Ref. 146), especially related to dysregulated phosphate metabolism.

## Directed acyclic graph

**Figure 3.**
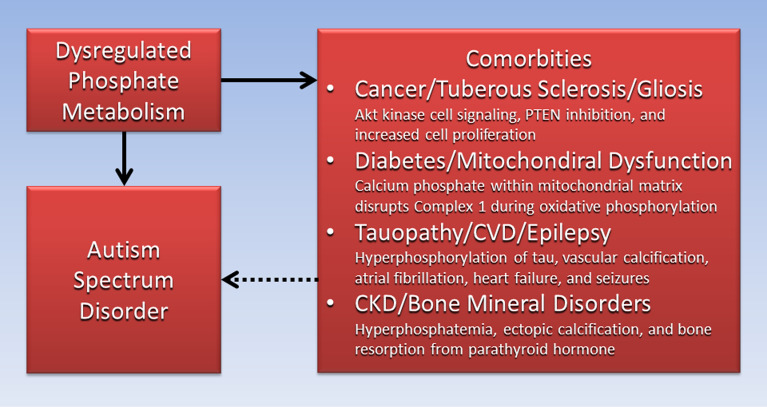
Dysregulated phosphate metabolism is causatively linked to both autism spectrum disorder and comorbidities associated with autism spectrum disorder.

Figure 3 is a directed acyclic graph (DAG), often used in epidemiological and clinical research to illustrate causal pathways and mediating factors that help explain the association of outcomes with exposures to risk factors (Ref. 147). The DAG in Figure 3 shows that dysregulated phosphate metabolism is a potential mediating factor causatively linking (solid arrows) associations between ASD and comorbidities reviewed in this paper (dotted arrow). The DAG helps identify new pathways mediated by dysregulated phosphate metabolism for future research in the cause and prevention of ASD and associated comorbidities.

## Conclusion

Evidence reviewed in this paper suggests that the effects of dysregulated phosphate metabolism and phosphate toxicity in ASD appear across the full lifespan, from the maternal stages of early development, throughout childhood and into adulthood. Dysregulated phosphate is proposed to stimulate gliosis of the brain in ASD, which disrupts neuro-circuitry by blocking axon extension and shortening neurite outgrowth. Children with ASD were found to have a wide variety of immune and neuroinflammatory responses. The rapid increase in ASD prevalence coincidences with the general population's increased consumption of ultraprocessed foods that are high in phosphate additives. Dysregulated phosphate metabolism is also causatively linked to comorbidities associated with ASD such as abnormal proliferation of cells, Akt kinase cell signalling and PTEN inhibition in cancer and tuberous sclerosis. Comorbidities also include calcium phosphate precipitation in mitochondrial dysfunction, impaired hormone secretion in diabetes, seizures in epilepsy, inflammatory and immune responses in CKD, obesity linked to excessive intake of ultraprocessed food, hyperphosphorylation in tauopathy, atrial fibrillation and heart failure in CVD, and bone resorption and ectopic calcification in bone mineral disorders.

Although other environmental factors and toxic conditions besides phosphate toxicity may contribute to ASD, findings implicating dysregulated phosphate metabolism in ASD warrant further investigation. The evidence presented in the present paper offers novel insights and directions for future research linking dysregulated phosphate metabolism with the aetiology of ASD. Additionally, future studies examining the effect of reduced dietary phosphorus in ASD are warranted.
